# The Correlation Between Survival Benefit of Preoperative Radiotherapy and Pretreatment Carcinoembryonic Antigen Level in Locally Advanced Rectal Cancer

**DOI:** 10.3389/fonc.2021.735882

**Published:** 2021-10-07

**Authors:** Lei Wang, Xiaohong Zhong, Huaqin Lin, Lingdong Shao, Gang Chen, Junxin Wu

**Affiliations:** ^1^ Department of Radiation Oncology, Fujian Medical University Cancer Hospital, Fujian Cancer Hospital, Fuzhou, China; ^2^ Department of Pathology, Fujian Medical University Cancer Hospital, Fujian Cancer Hospital, Fuzhou, China

**Keywords:** locally advanced rectal cancer, preoperative radiotherapy, carcinoembryonic antigen, SEER, biomarker

## Abstract

**Background:**

Preoperative radiotherapy followed by radical surgery is the standard treatment for locally advanced rectal cancer; however, its long-term survival benefit remains controversial. This study aimed to determine the relationship between pretreatment carcinoembryonic antigen (CEA) levels and the long-term prognosis of preoperative radiotherapy in locally advanced rectal cancer (LARC) patients.

**Methods:**

Data of LARC patients who underwent surgery between 2011 and 2015 were identified from the Surveillance, Epidemiology, and End Results (SEER) database, and patients were accordingly divided into surgery (S) group and radiotherapy followed by surgery (RT+S) group. The primary outcomes were cancer-specific survival (CSS) and cancer-specific mortality (CSM). CSS was evaluated using Kaplan-Meier analysis, while CSM was evaluated using a competitive risk model. Subgroup analysis was also conducted, which was stratified by pretreatment CEA levels.

**Results:**

A total of 2,760 patients were eligible for this study, including 350 (12.7%) patients in the S group and 2,410 (87.3%) in the RT+S group. There were no significant differences in the CSS and CSM rates at 1, 3, and 4 years between the S and RT+S groups before and after PSM (all *p* > 0.05). Pretreatment CEA levels were independently associated with CSS and CSM after adjusting for age, sex, stage, pathological factors, and treatment factors (all *p* < 0.05). Subgroup analysis showed that preoperative radiotherapy would benefit patients with elevated CEA in terms of CSS and CSM (both *p* < 0.05) but not those patients with normal CEA (both *p* > 0.05). Further analysis showed that preoperative radiotherapy was an independent protective factor for CSS and CSM in patients with elevated CEA levels (both *p* < 0.05).

**Conclusions:**

Pretreatment CEA level may be considered a potential biomarker to screen LACR patients who would benefit from preoperative radiotherapy in terms of long-term prognosis.

## Introduction

Preoperative radiotherapy, either long-course radiotherapy (LCRT) or short-course radiotherapy (SCRT), is the standard neoadjuvant strategy for locally advanced rectal cancer (LARC) (1, 2). In the recent decade, the proportion of LARC patients receiving preoperative radiotherapy has been as high as 59.8% in the USA (3), although direct surgery is still preferred in some areas, such as Japan (4). With the advent of preoperative radiotherapy, the rate of sphincter preservation is increasing, this being mainly due to the significant downstaging effect (5, 6). However, as a hard endpoint of treatment, the long-term survival benefit of preoperative radiotherapy remains controversial, regardless of LCRT and SCRT (7–11).

Carcinoembryonic antigen (CEA) is a routine screening and diagnostic index of colorectal cancer and is a widely used screening marker for postoperative recurrence (12–15). CEA levels, both before and after surgery, have been identified as important risk factors for long-term prognosis as well as for dynamic changes in CEA levels (16, 17). Moreover, CEA levels have been found to be associated with the response rate of neoadjuvant treatment (12, 18). However, there are no reports on the use of CEA in guiding the management of preoperative radiotherapy in terms of long-term prognosis. In the current study, we selected LARC patients diagnosed between 2011 and 2015 in the Surveillance, Epidemiology, and End Results (SEER) database to identify the long-term survival benefit of preoperative radiotherapy and to determine the relationship between the pretreatment CEA level and the prognosis of patients receiving preoperative radiotherapy.

## Materials and Methods

### Ethics Statement

Since we gained an official permit to access the research data of the SEER database (ID: 22032-Nov2019) and all the analyses in the current study were conducted under the rules of the SEER database, neither informed consent nor ethical approval was required for this study.

### Data Source

Patients aged 18 years or older who were diagnosed with rectal adenocarcinoma by pathology (2011 to 2015) were identified using the World Health Organization’s International Classification of Disease (ICD), 3rd edition (8140, 8144, 8210, 8211, 8213, 8221, 8255, 8261, 8263). Data on age, sex, marital status, insurance, pretreatment serum CEA level, tumor size, tumor differentiation, tumor deposits (TD), perineural invasion (PNI) status, circumferential resection margin (CRM), number of dissected lymph nodes (LND), tumor stage, node stage, surgery, radiation before surgery, chemotherapy, and survival (survival time and cause of death) were extracted from the SEER database.

### Patient Selection

Patients were eligible if they: (1) underwent radical surgery and were diagnosed with rectal cancer by pathology, (2) staged at T_3–4_N_any_M_0_ or T_any_N_+_M_0_, and (3) received chemotherapy. Patients were excluded from this study if they met one of the following criteria: (1) receipt of postoperative radiotherapy, (2) multiple cancers, (3) survival month ≤1 month, or (4) unknown clinical data. Based on whether patients received preoperative radiotherapy or not, they were divided into surgery (S) and radiotherapy +surgery (RT+S) groups.

### Variable Definition and Stratification

Variables were categorized according to the 8th American Joint Committee on Cancer guidelines or based on published studies: age at diagnosis (≤65 years, >65 years), sex (male or female), marital status (unmarried, married, other), insurance (no, yes), CEA level (normal, elevated), tumor size (≤5 cm, >5 cm), tumor differentiation (I/II, III/IV), TD (negative, positive), PNI (absent, present), CRM (negative, positive), number of LND (<12 or ≥12), stage (II, III), T stage (T1-2, T3, T4), N stage (N0, N1, N2), and survival (months).

### Outcome Definition

The endpoints of this study were cancer-specific survival (CSS) and cancer-specific mortality (CSM). CSS was defined as the time from the date of diagnosis to the date of death from rectal cancer or the latest follow-up. CSM was defined as cumulative mortality from the date of diagnosis to the date of death from rectal cancer or at the latest follow-up.

### Propensity Score Matching

Propensity score matching (PSM) analysis was performed to reduce selection bias. Briefly, baseline characteristics between the two groups were matched using the 1:1 nearest-neighbor matching method with a standard deviation of 0.2.

### Statistical Analyses

The chi-square (*χ* ^2^) test or Fisher’s test was used for comparisons between the two groups. The Kaplan–Meier (K-M) method was used for comparison of CSS analysis between the two groups using a log-rank test. A multivariate Cox regression model was used to identify the independent risk factors for CSS.

In the competitive-risk analysis, death from other causes was recognized as a competitive event of cancer-specific death. Gray’s test was used to determine the intergroup difference in the CSM, and the subdistribution proportional hazards model was used to perform multivariate analysis of CSM.

All statistical tests were conducted using RStudio (version 1.3.1073) in this study, including packages of xlsx, **Table 1**, survival, Survminer, MatchIt, cmprsk, and plyr. All tests were two-sided, and statistical significance was set at *p* < 0.05.

**Table 1:** Demographics and clinicopathologic characteristics of patients.

	Pre-PSM			After PSM		
	S (*N* = 350)	RT+S (*N* = 2,410)	*p*-Value	S (*N* = 331)	RT+S (*N* = 331)	*p*-Value
Age						
≤65 years	254 (72.6%)	1,758 (72.9%)	0.934	238 (71.9%)	248 (74.9%)	0.428
>65 years	96 (27.4%)	652 (27.1%)		93 (28.1%)	83 (25.1%)	
Sex						
Male	197 (56.3%)	1,532 (63.6%)	0.010	192 (58.0%)	196 (59.2%)	0.813
Female	153 (43.7%)	878 (36.4%)		139 (42.0%)	135 (40.8%)	
Marital status						
Unmarried	56 (16.0%)	470 (19.5%)	0.094	54 (16.3%)	74 (22.4%)	0.139
Married	231 (66.0%)	1,446 (60.0%)		216 (65.3%)	198 (59.8%)	
Other	63 (18.0%)	494 (20.5%)		61 (18.4%)	59 (17.8%)	
Insurance						
No	7 (2.0%)	100 (4.1%)	0.072	7 (2.1%)	5 (1.5%)	0.771
Yes	343 (98.0%)	2,310 (95.9%)		324 (97.9%)	326 (98.5%)	
CEA						
Normal	224 (64.0%)	1,372 (56.9%)	0.015	213 (64.4%)	207 (62.5%)	0.687
Elevated	126 (36.0%)	1,038 (43.1%)		118 (35.6%)	124 (37.5%)	
Tumor size						
≤5 cm	232 (66.3%)	1,589 (65.9%)	0.945	219 (66.2%)	235 (71.0%)	0.209
>5 cm	118 (33.7%)	821 (34.1%)		112 (33.8%)	96 (29.0%)	
Tumor differentiation						
Grade I/II	295 (84.3%)	2,153 (89.3%)	0.007	279 (84.3%)	282 (85.2%)	0.829
Grade III/IV	55 (15.7%)	257 (10.7%)		52 (15.7%)	49 (14.8%)	
TD						
Negative	271 (77.4%)	2,100 (87.1%)	<0.001	263 (79.5%)	251 (75.8%)	0.305
Positive	79 (22.6%)	310 (12.9%)		68 (20.5%)	80 (24.2%)	
PNI						
Absent	285 (81.4%)	2,120 (88.0%)	<0.001	271 (81.9%)	267 (80.7%)	0.765
Present	65 (18.6%)	290 (12.0%)		60 (18.1%)	64 (19.3%)	
CRM						
Negative	322 (92.0%)	2,227 (92.4%)	0.873	306 (92.4%)	300 (90.6%)	0.485
Positive	28 (8.0%)	183 (7.6%)		25 (7.6%)	31 (9.4%)	
Number of LND						
<12	44 (12.6%)	632 (26.2%)	<0.001	42 (12.7%)	43 (13.0%)	1.000
≥12	306 (87.4%)	1,778 (73.8%)		289 (87.3%)	288 (87.0%)	
Stage						
II	84 (24.0%)	843 (35.0%)	<0.001	83 (25.1%)	81 (24.5%)	0.928
III	266 (76.0%)	1,567 (65.0%)		248 (74.9%)	250 (75.5%)	
T stage						
T1–2	76 (21.7%)	137 (5.7%)	<0.001	66 (19.9%)	57 (17.2%)	0.483
T3	236 (67.4%)	2,030 (84.2%)		230 (69.5%)	244 (73.7%)	
T4	38 (10.9%)	243 (10.1%)		35 (10.6%)	30 (9.1%)	
N stage						
N0	84 (24.0%)	843 (35.0%)	<0.001	83 (25.1%)	81 (24.5%)	0.952
N1	195 (55.7%)	1,245 (51.6%)		187 (56.5%)	186 (56.2%)	
N2	71 (20.3%)	322 (13.4%)		61 (18.4%)	64 (19.3%)	

CEA, carcinoembryonic antigen; TD, tumor deposits; PNI, perineural invasion; CRM, circumferential resection margin; LND, dissected lymph nodes; S, surgery; RT, radiotherapy; PSM, propensity score matching.

## Results

### Patients’ Characteristics

A total of 2,760 patients were eligible for this study, including 350 (12.7%) patients in the S group and 2,410 (87.3%) in the RT+S group. The baseline characteristics between the S and RT+S groups were unparalleled, as depicted in **Table 1**. Briefly, the proportions of males, elevated CEA level, LND <12, and T3 in the RT+S group were all higher than those in the S group (all *p* < 0.05, **Table 1**), while the rates of tumor differentiation grades III/IV, TD, PNI, stage III, and N1/2 were lower in the RT+S group than in the S group (all *p* < 0.05, **Table 1**). However, the baseline characteristics between the two groups were comparable after 1:1 PSM (all *p* > 0.05, **Table 1**).

### Effect of Preoperative Radiotherapy on CSS and CSM in LARC Patients

**Figure 1:**
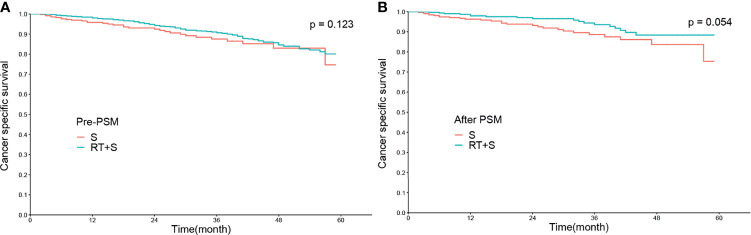
Cancer-specific survival of locally advanced rectal cancer before PSM **(A)** and after PSM **(B)**. S, surgery; RT, radiotherapy; PSM, propensity score matching.

**Figure 2:**
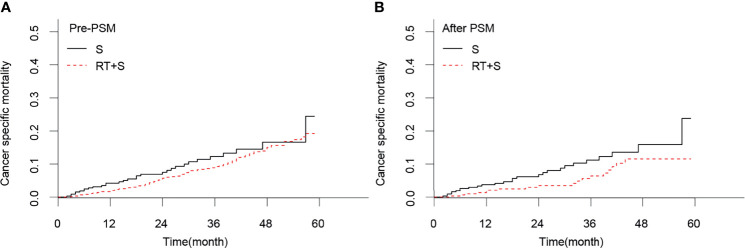
Cancer-specific mortality of locally advanced rectal cancer before PSM **(A)** and after PSM **(B)**. S, surgery; RT, radiotherapy; PSM, propensity score matching.

Before PSM, there were no significant differences in the CSS rates at 1, 3, and 4 years between the RT+S and S groups (98.02% *vs*. 95.78%, *p* = 0.078; 90.63% *vs*. 87.51%, *p* = 0.224; 84.57% *vs*. 82.94%, *p* = 0.374, respectively; **Figure 1A**), as well as the 1-, 3-, and 4-year CSM rates (1.97% *vs*. 4.20%, *p* = 0.068; 9.21% *vs*. 12.28%, *p* = 0.189; 15.03% *vs*. 16.61%, *p* = 0.364, respectively; **Figure 2A**). After PSM, the CSS rates at 1, 3, and 4 years in the RT+S group were higher than those in the S group, but there were still no statistical differences between the two groups (97.94% *vs*. 96.27%, *P* = 0.203; 93.58% *vs*. 88.64%, *p* = 0.134; 88.36% *vs*. 83.68%, *p* = 0.279, respectively; **Figure 1B**). Similar findings were observed in terms of the 1-, 3-, and 4-year CSM rates (2.05% *vs*. 3.72%, *p* = 0.194; 6.38% *vs*. 11.18%, *p* = 0.110; 11.53% *vs*. 15.88%, *p* = 0.255, respectively; **Figure 2B**).

### Effect of Pretreatment CEA Level on CSS and CSM in LARC Patients

In the matched cohort, time-dependent coefficient analysis showed a strong association between CEA and CSS (unadjusted hazard ratio (HR) = 2.50, 95% confidence interval (CI) = 1.38–4.51, *p* = 0.002). Age-adjusted HR for CSS among elevated CEA patients compared with normal CEA patients was 2.48 (95% CI = 1.37–4.49, *p* = 0.003). Further adjustments for sex (model 2), stage (model 3), pathological factors (model 4), and treatment factors (model 5) showed similar results (all *p* < 0.05, **Table 2**). Furthermore, these associations were not attenuated after adjustment for all five factor groups (model 6). Likewise, pretreatment CEA level was also found to be an independent risk factor of CSM regardless of the model (all *p* < 0.05, **Table 2**).

**Table 2:** Effect of pretreatment CEA level on CSS and CSM in LARC patients.

Model	CSS		CSM	
	HR (95% CI)	*p*-Value	HR (95% CI)	*p*-Value
Unadjusted	2.50 (1.38, 4.51)	0.002	2.44 (1.36, 4.38)	0.003
Model 1^a^	2.48 (1.37, 4.49)	0.003	2.39 (1.33, 4.30)	0.004
Model 2^b^	2.59 (1.43, 4.69)	0.002	2.53 (1.41, 4.54)	0.002
Model 3^c^	2.41 (1.33, 4.37)	0.004	2.35 (1.32, 4.21)	0.004
Model 4^d^	1.91 (1.03, 3.53)	0.039	1.87 (1.01, 3.45)	0.046
Model 5^e^	2.58 (1.43, 4.67)	0.002	2.51 (1.39, 4.53)	0.002
Model 6^f^	2.03 (1.08, 3.82)	0.028	1.95 (1.02, 3.72)	0.043

CEA, carcinoembryonic antigen; CSS, cancer-specific survival; CSM, cancer-special mortality; HR, hazard ratio; CI, confidence interval; TD, tumor deposits; PNI, perineural invasion; CRM, circumferential resection margin; LND, dissected lymph nodes.

a Adjusted for age.

b Adjusted for sex.

c Adjusted for stage.

d Adjusted for pathological factors (tumor size, tumor differentiation, TD, PNI, CRM).

e Adjusted for treatment factors (number of LND, radiotherapy).

f Adjusted for age, sex, stage, pathological factors, and treatment factors.

### Effect of Preoperative Radiotherapy on CSS and CSM in Normal CEA Subgroup

**Figure 3:**
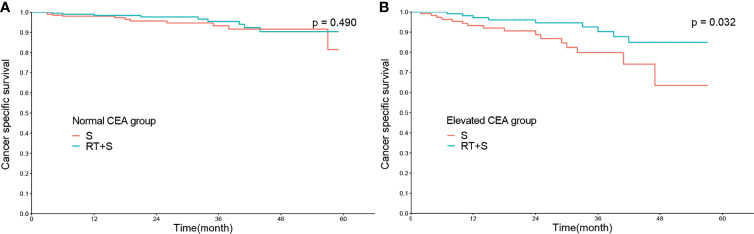
Cancer-specific survival of locally advanced rectal cancer in normal CEA group **(A)** and elevated CEA group **(B)**. CEA, carcinoembryonic antigen; S, surgery; RT, radiotherapy.

**Figure 4:**
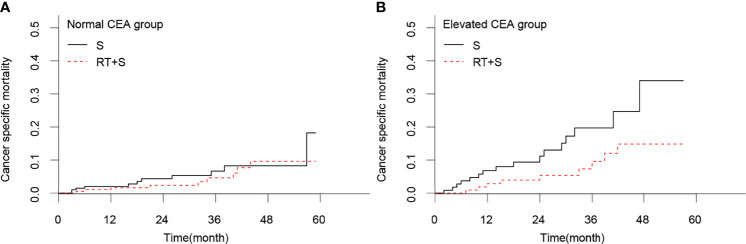
Cancer-specific mortality of locally advanced rectal cancer in normal CEA group **(A)** and elevated CEA group **(B)**. CEA, carcinoembryonic antigen; S, surgery; RT, radiotherapy.

In the matched cohort, 420 patients had normal CEA levels, including 213 patients in the S group and 207 in the RT+S group. Of note, there were no significant differences between the S and RT+S groups in terms of baseline characteristics (all *p* < 0.05, **Table 3**). K-M survival analysis showed that there was no significant difference in the median CSS between the two groups (HR = 0.73, 95% CI = 0.30–1.77, *p* = 0.490, **Figure 3A**). A similar finding was observed in CSM (HR = 0.74, 95% CI = 0.31–1.78, *p* = 0.500, **Figure 4A**).

**Table 3:** Demographics and clinicopathologic characteristics of patients with normal CEA level.

	S (*N* = 213)	RT+S (*N* = 207)	*p*-Value
Age			
≤65 years	157 (73.7%)	160 (77.3%)	0.459
>65 years	56 (26.3%)	47 (22.7%)	
Sex			
Male	126 (59.2%)	130 (62.8%)	0.505
Female	87 (40.8%)	77 (37.2%)	
Marital status			
Unmarried	32 (15.0%)	42 (20.3%)	0.309
Married	144 (67.6%)	127 (61.4%)	
Other	37 (17.4%)	38 (18.3%)	
Insurance			
No	5 (2.3%)	2 (1.0%)	0.469
Yes	208 (97.7%)	205 (99.0%)	
Tumor size			
≤5 cm	151 (70.9%)	157 (75.8%)	0.300
>5 cm	62 (29.1%)	50 (24.2%)	
Tumor differentiation			
Grade I/II	178 (83.6%)	174 (84.1%)	0.997
Grade III/IV	35 (16.4%)	33 (15.9%)	
TD			
Negative	179 (84.0%)	166 (80.2%)	0.368
Positive	34 (16.0%)	41 (19.8%)	
PNI			
Absent	185 (86.9%)	180 (87.0%)	1.000
Present	28 (13.1%)	27 (13.0%)	
CRM			
Negative	200 (93.9%)	193 (93.2%)	0.939
Positive	13 (6.1%)	14 (6.8%)	
Number of LND			
<12	22 (10.3%)	22 (10.6%)	1.000
≥12	191 (89.7%)	185 (89.4%)	
Stage			
II	59 (27.7%)	58 (28.0%)	1.000
III	154 (72.3%)	149 (72.0%)	
T stage			
T1–2	58 (27.2%)	50 (24.2%)	0.647
T3	137 (64.3%)	142 (68.6%)	
T4	18 (8.5%)	15 (7.2%)	
N stage			
N0	59 (27.7%)	58 (28.0%)	0.957
N1	123 (57.7%)	117 (56.5%)	
N2	31 (14.6%)	32 (15.5%)	

TD, tumor deposits; PNI, perineural invasion; CRM, circumferential resection margin; LND, dissected lymph nodes; S, surgery; RT, radiotherapy.

### Effect of Preoperative Radiotherapy on CSS and CSM in Elevated CEA Subgroup

In the matched cohort, 242 patients were present with pre-treatment elevated CEA levels, including 118 patients in the S group and 124 in the RT+S group. Likewise, no significant differences were observed between the S and RT+S groups in terms of baseline characteristics (all *p* < 0.05, **Table 4**). The pooled HR for the median CSS was in favor of the RT+S group as compared with the S group (HR = 0.41, 95% CI = 0.18–0.92, *p* = 0.032, **Figure 3B**), with elevated survival rates at 1, 3, and 4 years (97.12% *vs*. 93.22%, 90.24% *vs*. 79.79%, 84.91% *vs*. 63.51%, respectively). Similar differences were also observed between the two groups (HR = 0.42, 95% CI = 0.19-0.94, *p* = 0.032; **Figure 4B**). Furthermore, multivariate analysis in the elevated CEA subgroup showed that preoperative radiotherapy was an independent protective factor for CSS and CSM (CSS: HR = 0.36, 95% CI = 0.15–0.83, *p* = 0.017; CSM: HR = 0.41, 95% CI = 0.18–0.94, *p* = 0.036, respectively; **Table 5**).

**Table 4:** Demographics and clinicopathologic characteristics of patients with elevated CEA level.

	S (*N* = 118)	RT+S (*N* = 124)	*p*-Value
Age			
≤65 years	81 (68.6%)	88 (71.0%)	0.800
>65 years	37 (31.4%)	36 (29.0%)	
Sex			
Male	66 (55.9%)	66 (53.2%)	0.769
Female	52 (44.1%)	58 (46.8%)	
Marital status			
Unmarried	22 (18.7%)	32 (25.8%)	0.385
Married	72 (61.0%)	71 (57.3%)	
Other	24 (20.3%)	21 (16.9%)	
Insurance			
No	2 (1.7%)	3 (2.4%)	1.000
Yes	116 (98.3%)	121 (97.6%)	
Tumor size			
≤5 cm	68 (57.6%)	78 (62.9%)	0.479
>5 cm	50 (42.4%)	46 (37.1%)	
Tumor differentiation			
Grade I/II	101 (85.6%)	108 (87.1%)	0.878
Grade III/IV	17 (14.4%)	16 (12.9%)	
TD			
Negative	84 (71.2%)	85 (68.5%)	0.759
Positive	34 (28.8%)	39 (31.5%)	
PNI			
Absent	86 (72.9%)	87 (70.2%)	0.744
Present	32 (27.1%)	37 (29.8%)	
CRM			
Negative	106 (89.8%)	107 (86.3%)	0.516
Positive	12 (10.2%)	17 (13.7%)	
Number of LND			
<12	20 (16.9%)	21 (16.9%)	1.000
≥12	98 (83.1%)	103 (83.1%)	
Stage			
II	24 (20.3%)	23 (18.5%)	0.850
III	94 (79.7%)	101 (81.5%)	
T stage			
T1–2	8 (6.8%)	7 (5.6%)	0.795
T3	93 (78.8%)	102 (82.3%)	
T4	17 (14.4%)	15 (12.1%)	
N stage			
N0	24 (20.3%)	23 (18.6%)	0.939
N1	64 (54.2%)	69 (55.6%)	
N2	30 (25.5%)	32 (25.8%)	

TD, tumor deposits; PNI, perineural invasion; CRM, circumferential resection margin; LND, dissected lymph nodes; S, surgery; RT, radiotherapy.

**Table 5:** Multivariate analysis on CSS and CSM of patients with elevated CEA level.

Variable	CSS		CSM	
	HR (95% CI)	*p*-Value	HR (95% CI)	*p*-Value
Age (≤65 *vs*. >65 years)	3.89 (1.66, 9.1)	0.002	3.33 (1.47, 7.56)	0.004
Sex (female *vs*. male)	–	–	–	–
Marital status	–	–	–	–
Tumor size (≤5 *vs*. >5 cm)	–		–	–
Tumor differentiation(III/IV *vs*. I/II)	–	–	–	–
TD (positive *vs*. negative)	–	–	–	–
PNI (present *vs*. absent)	1.35 (0.55, 3.31)	0.514	1.28 (0.52, 3.15)	0.590
CRM (positive *vs*. negative)	4.21 (1.66, 10.68)	0.002	3.91 (1.43, 10.67)	0.008
Number of LND (≥12 *vs*. <12)	–	–	–	–
Stage (III *vs*. II)	–	–	–	–
Radiotherapy (yes *vs*. no)	0.36(0.15,0.83)	0.017	0.41(0.18,0.94)	0.036

CSS, cancer-specific survival; CSM, cancer-specific mortality; CEA, carcinoembryonic antigen; HR, hazard ratio; CI, confidence interval; TD, tumor deposits; PNI, perineural invasion; CRM, circumferential resection margin; LND, dissected lymph nodes.

## Discussion

The question whether preoperative radiotherapy can bring long-term survival benefit to LARC patients has been troubling the minds of surgeons and radiotherapists for a long time (7–11). In the current study, we found that pretreatment CEA level was a robust risk factor for prognosis after adjusting for confounding factors in different models. Furthermore, we also found that only a subgroup of LARC patients with elevated pretreatment CEA levels will benefit from preoperative radiotherapy in terms of CSS and CSM.

Preoperative radiotherapy followed by radical surgery has been preferred prevalently mainly due to its advantage on downstaging, pathological complete response (pCR), sphincter preservation and superior to adjuvant radiotherapy in prevention of local recurrence (19–21), although it can (1) increase the risk of surgical complications (22, 23), (2) bring radiation-related toxicity (24, 25), and (3) cannot improve the long-term prognosis of LARC patients when compared with adjuvant radiotherapy (20, 26, 27). In this study, 87.3% of patients received preoperative radiotherapy from 2011 to 2015 in the SEER database with a satisfactory 4-year CSS rate of 88.36%, indicating the importance of standardization treatment. Furthermore, in the recent decade, more neoadjuvant strategies have been explored with inspiring results in trials of FOWARC, RAPDIO, PRODIGEL 23, and IWWD, which attach more importance to preoperative radiotherapy (7, 9, 28, 29). The pCR rate is reported to range from 16.1% to 30% (9, 30–32), and patients with pCR generally have a better prognosis (33–35). However, the long-term survival benefit of preoperative radiotherapy has rarely been identified in previous reports (**Table 6**) (5–7, 9, 21, 28, 30, 36–38). In this study, we found that preoperative radiotherapy did not improve CSS in LARC patients before and after PSM (both *p* > 0.05). Additionally, we applied a competitive risk model to identify the true effect size of preoperative radiotherapy on long-term prognosis, since the rates of competition events were as high as 25% before PSM and as high as 21% after PSM. However, the results of the competitive risk model were highly consistent with the results of traditional K-M analysis, which indicated that noncancer-related mortality may have little effect on the conclusion of the study. Nonetheless, preoperative radiotherapy could not benefit LARC patients in terms of CSM before and after PSM (both *p* > 0.05). The reasons for this occurrence may be as follows: (1) in the era of neoadjuvant treatment followed by total mesorectal excision (TME), distant metastasis but not local recurrence is the decisive factor for long-term prognosis (7, 39); otherwise, (2) in the matched cohort, apparent survival differences are observed between the RT+S and S groups both in terms of CSS and CSM but with a margin *p*-value (*p* = 0.054, *p* = 0.059, respectively), which indicates that a larger sample size may be needed to avoid false-negative results.

**Table 6:** Overall survival of locally advanced rectal cancer patients associated with neoadjuvant radiotherapy in phase III RCTs.

Trial	Recruitment time	Sample size	Study design	OS	*p*-Value
Dutch TME trial^37^	1996–1999	1,805	Preoperative SCRT+TME *vs*. TME alone	62.2% *vs*. 61.9%^a^	0.86
German CAO/ARO/AIO-094^21^	1995–2002	799	Preoperative *vs*. postoperative LCRT	76% *vs*. 74%^a^	0.80
Polish^30^	1999–2002	302	Preoperative SCRT *vs*. LCRT	67.2% *vs*. 66.2%^b^	0.96
NSABP-R03^38^	2004–2010	254	Preoperative *vs*. postoperative LCRT	74.5% *vs*. 65.6%^a^	0.065
German CAO/ARO/AIO-04^6^	2006–2010	1,236	Preoperative LCRT: 5-FU+oxaliplatin *vs*. 5-FU	88.7% *vs*. 88.0%^c^	NA
Stockholm III^5^	1998–2013	385	Preoperative SCRT *vs*. SCRT-delay *vs*. LCRT-delay	73% *vs*. 76% *vs*. 78%^a^	NA
Polish II^36^	2008–2014	515	Preoperative SCRT+CCT *vs*. LCRT	73% *vs*. 65%^c^	0.046
FOWARC^28^	2010–2015	330	Preoperative LCRT: mFOLFOX *vs*. 5FU	89.1% *vs*. 91.3%^c^	0.96
RAPIDO^7^	2011–2016	912	Preoperative SCRT+CCT *vs*. LCRT	83% *vs*. 81%^d^	NA
PRODIGE 23^9^	2012–2017	460	Preoperative ICT+LCRT *vs*. LCRT	87.7% *vs*. 90.8%^c^	0.08

RCTs, randomized clinical trials; OS, overall survival; SCRT, short-course radiotherapy; LCRT, long-course radiotherapy; 5-FU, fluorouracil; FOLFOX, folinic acid, 5-FU, and oxaliplatin; TME, total mesorectal excision; delay, radiotherapy with surgery after 4–8 weeks; CCT, consolidation chemotherapy; ICT, induction chemotherapy; NA, not available.

a Five-year OS.

b Four-year OS.

c Three-year OS.

d 4.6-year OS.

As a tumor-associated antigen, CEA has been used as a specific marker for the early diagnosis of colorectal cancer, however, its specificity is far from satisfactory (40, 41). In the current study, only 1,164 (42.6%) patients presented with elevated pre-treatment CEA levels. Nevertheless, the pretreatment CEA level was found to be an independent risk factor for both CSS and CSM, and it was reconfirmed in different models by adjusting for age, sex, tumor characteristics, and treatment factors (all *p* < 0.05), which indicated that pretreatment CEA level was a robust prognostic factor of long-term survival, and there may be an interaction between pre-treatment CEA level and preoperative treatment on long-term prognosis.

The relationship between CEA level and preoperative radiotherapy has been explored, however, pretreatment CEA level is not an indicator of preoperative radiotherapy. Pretreatment CEA level has been identified as a predictive biomarker of neoadjuvant treatment response, as well as post-treatment CEA levels and dynamic changes in CEA levels (18). Furthermore, CEA is associated with radiation sensitivity; tumors with normal CEA are sensitive to radiation, while tumors with elevated CEA levels are resistant to radiation (12). In the current study, subgroup analysis stratified by pretreatment CEA level showed that preoperative radiotherapy would only benefit patients with elevated CEA levels. The reasons for this may be as follows: (1) preoperative radiotherapy could reduce the risk of local recurrence, which is an important risk factor for long-term prognosis; (2) the compliance of patients with elevated CEA is higher than those with normal CEA, who are much more likely to receive a full course of chemotherapy; and (3) more intensive postoperative monitoring would be conducted in patients with elevated CEA levels, indicating a more timely intervention for early recurrence/metastasis. This finding suggested that pretreatment CEA could also be used as a potential biomarker to screen patients who would enjoy the long-term survival benefit of preoperative radiotherapy.

However, there are several limitations to the current study. First, selection bias is difficult to avoid in a retrospective analysis, although a well-designed PSM was conducted in our study. Second, data on preoperative radiotherapy, including clinical target volume and radiation regimen, are unavailable in the SEER database, which would weaken the conclusion of the current study. Third, data on chemotherapy, such as regimen and courses, are also unavailable, which is one of the most important risk factors for long-term prognosis. Hence, in the present study, we excluded all those patients who had not received adjuvant chemotherapy to decrease the effect of adjuvant chemotherapy on long-term prognosis. Finally, the receipt rate of preoperative radiotherapy varies from region to region, which indicates that our conclusion needed further validation using either data outside of the USA or multicenter randomized clinical trials accordingly.

## Conclusion

Based on our results, we conclude that pretreatment CEA level may be considered a potential biomarker to screen LACR patients who would benefit from preoperative radiotherapy in terms of long-term prognosis.

## Data Availability Statement

The dataset analyzed in this study from SEER can be obtained from: https://seer.cancer.gov/data/.

## Ethics Statement

Ethical review and approval was not required for the study on human participants in accordance with the local legislation and institutional requirements. Written informed consent for participation was not required for this study in accordance with the national legislation and the institutional requirements.

## Author Contributions

LW, XZ, HL, LS, GC, and JW contributed to conception and design. XZ and HL conducted data collection and analyzed the data. LW, XZ, and HL interpreted the data. LW, XZ, and HL drafted the manuscript. LS, GC, and JW contributed to the critical revision of the manuscript. All authors contributed to the article and approved the submitted version.

## Funding

This research was funded by the Science and Technology Program of Fujian Province, China (No. 2019L3018 and 2019YZ016006); the Fujian Province Finance Department Project (No. (2019)827).

## Conflict of Interest

The authors declare that the research was conducted in the absence of any commercial or financial relationships that could be construed as a potential conflict of interest.

## Publisher’s Note

All claims expressed in this article are solely those of the authors and do not necessarily represent those of their affiliated organizations, or those of the publisher, the editors and the reviewers. Any product that may be evaluated in this article, or claim that may be made by its manufacturer, is not guaranteed or endorsed by the publisher.
